# Modulation of tocotrienol’s bone effects by osteocytes: a perspective

**DOI:** 10.3389/fphar.2026.1773544

**Published:** 2026-02-19

**Authors:** Nurul Nabihah Zahanordin, Pei Yuen Ng, Kok-Yong Chin

**Affiliations:** 1 Department of Pharmacology, Faculty of Medicine, Universiti Kebangsaan Malaysia, Cheras, Malaysia; 2 Centre of Drug Delivery Research, Faculty of Pharmacy, Universiti Kebangsaan Malaysia Kuala Lumpur Campus, Kuala Lumpur, Malaysia

**Keywords:** bone ageing, osteoblast, osteocyte, oxidative stress, tocotrienol

## Abstract

Osteocytes function as central regulators of skeletal health by acting as mechanosensors that control bone remodelling mediated by osteoblasts and osteoclasts. Disrupted osteocyte function, often driven by oxidative stress and linked to ageing and osteoporosis, contributes to pathological bone remodelling. Tocotrienols (TTs), a family of vitamin E, are intensively investigated for their bone-protective effects, with mechanisms that involve reducing intracellular reactive oxygen species, enhancing antioxidant defences, and modulating signalling pathways of bone remodelling. Preliminary studies suggest that TTs exert protective and anabolic effects by influencing osteocytes, including shielding them from oxidative damage. *In vivo* models using ovariectomised or metabolic syndrome rats demonstrated that TT supplementation modulated key osteocyte-secreted factors, including sclerostin, dentin matrix protein 1, Dickkopf-related protein 1, fibroblast growth factor 23, and receptor activator of nuclear factor κB ligand. However, the current evidence is limited by the use of models that may not fully represent degenerative osteoporosis, restricted dose-dependent studies, and the challenge of real-time *in vivo* monitoring. This perspective summarises the reported effects of TTs on osteocytes’ function and emphasises the critical need for future research to employ more representative animal models, advanced imaging techniques, and complex 3D co-culture or bone explant systems to accurately define the mechanism of action of TTs and their resulting functional outcomes on overall bone quality.

## 1 Introduction

Osteoporosis is a bone disease characterised by degenerative changes in bone quantity (bone mass) and quality (microstructure) associated with ageing, leading to a decline in bone strength and an increased risk of fractures (Chin et al., 2022). It is an increasingly important healthcare issue in the context of global societal ageing, given the healthcare burden associated with fragility fractures (GBD, 2019 Fracture collaborators, 2021). Both sexes are affected by osteoporosis; postmenopausal women are disproportionately affected by the disease (Liang et al., 2025), but men suffer from a higher mortality post-fracture (Larsen et al., 2025). Various pharmacology interventions have been designed to improve bone mass and reduce the risk of fractures, each with its advantages and side effects (Larsen et al., 2025).

Antiosteoporosis drugs primarily target the bone remodelling processes mediated by osteoblasts (bone-forming cells) and osteoclasts (bone-resorbing cells) (Elahmer et al., 2024). Recently, the most abundant bone cells, osteocytes, have also been identified as an avenue for intervention (Pathak et al., 2020). Osteocytes play a significant role in maintaining skeletal health. They are terminally differentiated from osteoblasts, which become embedded within the mineralised extracellular matrix of bone and assume regulatory functions. They have an extensive lacuno-canalicular network that acts as mechanosensor (Robling and Bonewald, 2020). They sense mechanical stress and regulate the function of osteoblasts and osteoclasts to maintain bone remodelling and structural stability (Yan et al., 2020).

Osteocytes secrete regulatory molecules, such as receptor activator of nuclear factor kappa-B ligand (RANKL), osteoprotegerin (OPG), dentin matrix protein 1 (DMP1), sclerostin (SOST), and fibroblast growth factor-23 (FGF23) (Kitaura et al., 2020). RANKL is involved in osteoclastogenesis, while OPG counterbalances this effect by preventing excessive bone resorption (Divieti Pajevic and Krause, 2019). SOST maintains bone formation within physiological limits by suppressing Wnt/β-catenin signalling in osteoblasts, limiting their survival and activities (Chen et al., 2025). Additionally, FGF23 regulates phosphate metabolism, linking osteocyte activity to systemic mineral homeostasis (Kurpas et al., 2021). The current antibody-based therapies for osteoporosis target SOST (e.g., romosozumab) and RANKL (e.g., denosumab) to achieve their therapeutic potential (Pang et al., 2020; Wu et al., 2023).

Tocotrienols (TTs) are a natural product that has been investigated intensively for its bone-protective effects through multiple mechanisms, potentially involving osteocytes (Chin, 2024a; Chin, 2024b). TTs are a family of vitamin E with double bonds on the carbon tail. They are found mainly in palm oil, annatto bean and rice bran, along with other sources, including grains and grapes (Pang and Chin, 2019). TTs contribute to the reduction of intracellular reactive oxygen species (ROS), enhance cellular antioxidant defences, and regulate the RANKL/OPG balance. Furthermore, they are involved in protective signalling mechanisms, notably the phosphoinositide 3-kinase/protein kinase B-nuclear factor erythroid 2-related factor 2 (NRF2) signalling pathway (Casati et al., 2020). TTs also play a significant role in suppressing the mevalonate pathway while also promoting the increased expression of osteoprotegerin (OPG) mRNA. This regulatory mechanism results in decreased production of RANKL. Consequently, this signalling cascade leads to diminished bone resorption, thereby enhancing protection against bone loss (Wong et al., 2019b; Ormsby et al., 2022).

Recent studies suggest that the therapeutic effects of TTs are mediated by osteocytes, but a structured review on this aspect has not been conducted. Thus, this review aims to summarise previous studies regarding the effects of TTs on osteocytes, focusing on their potential mechanisms of action, cellular targets, and effects on bone health. The limitations and prospects of this field are also put forward to guide future research.

## 2 Osteocytes

### 2.1 Osteocytes and bone remodelling

**FIGURE 1:**
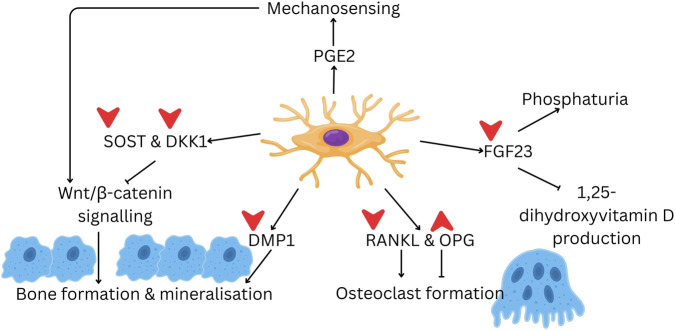
The multifaceted role of osteocytes in regulating bone remodelling. Osteocytes secrete signalling molecules, including PGE2, DMP1, FGF23, SOST, DKK1, RANKL, and OPG, which regulate various processes of bone remodelling, such as mechanosensing, mineral metabolism, bone formation, and osteoclast formation. The red arrows indicate potential targets of tocotrienol; up arrows indicate upregulation, and down arrows indicate downregulation. Abbreviation: DKK1, dickkopf-related protein 1; DMP1, dentin matrix protein 1; FGF23, fibroblast growth factor 23; OPG, osteoprotegerin; PGE2, prostaglandin E2; RANKL, receptor activator of nuclear factor κB ligand; SOST, sclerostin. (Drawn using Canva).

As stated earlier, osteocytes came from embedded osteoblasts within the lacuna-canalicular network. Osteocytes generate signals that are essential for controlling osteoclast formation and regulating phosphate metabolism, including proteins such as DMP1, SOST, and FGF23. Additionally, they serve as a significant source of RANKL (Wu et al., 2025). All these factors help osteocytes function as central regulators of bone remodelling (Figure 1).

The processes of mechanotransduction play a critical role in bone remodelling, which is regulated by osteocytes. Notably, the activation of Wnt/β-catenin signalling is partly mediated through Piezo one and integrin-linked pathways. This activation results in the suppression of SOST, thereby establishing a critical nexus between mechanical loading and the processes of bone formation and resorption (Wu et al., 2025). Furthermore, Wnt signalling in osteocytes increases the formation of osteoclasts by increasing RANKL expression through interactions with transforming growth factor-β (TGF-β) and Smad4 at the promoter region of RANKL (Ye et al., 2025).

Disruption of osteocyte function contributes to pathological remodelling. Processes such as apoptosis, ferroptosis or pyroptosis can disrupt the lacuna-canalicular network. This will lead to impairment of remodelling and repair capacity (Wu et al., 2025). Various osteocyte-targeted strategies show significant potential in restoring osteocyte function. These include anticytokine therapy, gene editing, stem cell interventions, and multi-omics approaches (Wu et al., 2025; Liu et al., 2025).

### 2.2 Pathophysiology: oxidative stress and inflammation in osteocytes

Oxidative stress is highly recognised as the central drive of skeletal deterioration. Elevated reactive oxygen species (ROS) will impair osteocyte homeostasis. Oxidative stress serves as a central mechanism in response to various insults, including glucocorticoids and homocysteine. These sources impaired osteocyte biology and activated maladaptive bone remodelling. These exposures upregulate the NOX family of NADPH oxidases, driving the cytokine expression and apoptosis in osteocyte-like cells (Notsu et al., 2019; Zhang et al., 2022). Mitochondrial dysfunction due to ageing or injury-related causes will also contribute to the upregulation of ROS, resulting from impaired mitophagy (Ardura et al., 2020).

The resulting reactive oxygen species activate a network of stress-responsive signalling pathways that reshape osteocyte survival, communication, and remodelling output. ROS stimulate the NLRP3 inflammasome, promoting caspase-1 activation, IL-1β maturation, and pyroptotic cell death (Kar et al., 2019). Activation of NF-κB and MAPK pathways leads to elevations in RANKL expression and the production of pro-resorptive cytokines (Marcucci et al., 2023). Oxidative stress also inhibits Wnt/β-catenin signalling by diverting β-catenin towards FOXO transcription factors, suppressing anabolic bone formation (Marcucci et al., 2023).

## 3 Tocotrienols

### 3.1 Tocotrienols: overview of skeletal protective mechanisms

Similar to the classic vitamin E, tocopherols (TPs), TTs can be categorised into four subtypes, such as alpha-, beta-, gamma-, and delta-homologues, differing by arrangement of the methyl group on the chromanol ring (Chin et al., 2013). TT and TF homologues exist in a mixture in natural sources (Aggarwal et al., 2010).

TTs exert both oxidative and non-oxidative actions on the skeletal system to achieve their anti-osteoporotic effects. TTs stimulate bone growth by facilitating the differentiation of osteoblasts and protecting these cells from oxidative stress (Abd Manan et al., 2012; Casati et al., 2020). It upregulates osteoblastogenic transcription factors, such as Runt-related transcription factor 2 (Runx2) and Osterix, thereby promoting the differentiation of osteoblasts (Wan Hasan et al., 2018). This action is potentially modulated by 3-hydroxy-3-methylglutaryl-coenzyme A reductase and GTPase activities (Wan Hasan et al., 2020). TTs also stimulate the Wnt signalling pathway to achieve pro-osteoblastogenesis effects (Xu et al., 2022). The anti-osteoclastogenic effects of TTs are mediated by the RANKL/OPG pathway and its anti-inflammatory effects. TT supplementation has been shown to modify the RANKL/OPG ratio and lower inflammation in the bone, favouring an anti-osteoclastogenesis milieu (Mohamad et al., 2021; Wong et al., 2018b).

### 3.2 Effects of tocotrienols on osteocytes

There are some preliminary studies on the effects of TT on osteocytes. Delta-TT protects osteocytes (MLO-Y4) from oxidative stress induced by tert-butyl hydroperoxide, a commonly used *in vitro* inducer of oxidative damage (Casati et al., 2020). Its cytoprotective properties combat oxidative stress by activating signalling pathways, such as PI3K/Akt and Nrf2. Through PI3K/Akt activation, delta-TT enhances cell survival signalling and promotes the phosphorylation of downstream targets that inhibit apoptosis (Casati et al., 2020). Simultaneously, through Nrf2 activation, it induces the expression of antioxidant defence genes, leading to increased production of protective enzymes. These coordinated actions result in a reduction of reactive oxygen species (ROS) levels by improving the glutathione (GSH)/glutathione disulfide (GSSG) ratio. GSH is a primary cellular antioxidant molecule, and maintaining a high GSH/GSSG ratio indicates a healthy cellular redox state (Vázquez-Meza et al., 2023).

However, in this study, the redox signalling was established in the osteoblast cell line rather than the osteocyte cell line. Although osteoblasts and osteocytes originate from the same lineage, they have distinct physiological roles and metabolic demands (Karthik and Guntur, 2021; Shen et al., 2022). Therefore, direct confirmation of these signalling mechanisms specifically in osteocytes would strengthen the conclusions about delta-TT’s protective effects in these cells.

The osteoporosis-reversing effects of emulsified and non-emulsified palm TT mixtures were investigated in a unique experimental design using ovariectomised rats (Mohamad et al., 2021). This study differed significantly from previous studies using TTs by employing a therapeutic rather than preventive approach, in which supplementation was initiated 2 months post-ovariectomy in a group of older rats (12 months old by the end of treatment). This experimental design is clinically relevant because it mimics the real-world scenario where osteoporosis treatment typically begins after significant bone loss has already occurred, rather than as a preventive measure.

The results showed no significant changes in the number of osteocytes and the number of empty lacunae of trabecular bone after a 2-month treatment (Mohamad et al., 2021). The lack of change in osteocyte numbers and empty lacunae suggests that the relatively short treatment duration (2 months) in rats with established, advanced bone loss may not have been sufficient to demonstrate recovery of the osteocyte population. Osteocyte turnover is a slow process; thus, the restoration of osteocyte networks takes considerable time (Lerebours and Buenzli, 2016). Empty lacunae represent sites of osteocyte death, and their persistence indicates ongoing or incompletely reversed bone deterioration.

Despite the lack of changes in osteocyte parameters, both emulsified and non-emulsified palm TT formulations significantly reduced skeletal SOST protein expression levels (Mohamad et al., 2021). This reduction in SOST expression is mechanistically important because a lower level would allow activation of Wnt/beta-catenin signalling in osteoblasts, potentially promoting bone formation (Delgado-Calle et al., 2017). This molecular change could represent an early adaptive response that precedes observable changes in bone structure and cell populations. This observation suggests that TTs can modulate osteocyte gene expression even in established osteoporosis, which may translate into improved bone formation with longer treatment duration.

The observation that both emulsified and non-emulsified palm TT produced similar effects on SOST expression suggests that the bioactive TT compounds themselves, rather than the delivery vehicle, are responsible for this molecular effect. The reduction in SOST represents a favourable shift in the bone remodelling environment that could potentially lead to increased bone formation if treatment were continued for a longer period.

In a subsequent study using a preventive model, ovariectomised rats were supplemented with emulsified and non-emulsified palm TT mixtures for 10 weeks, with treatment initiated 1 week after ovariectomy (Ekeuku et al., 2025). This preventive approach differs from the therapeutic approach described in the previous study, as treatment begins before substantial bone loss occurs, allowing assessment of TTs’ ability to prevent rather than reverse osteoporosis.

The results showed a significantly higher number of trabecular and cortical osteocytes than in both the palm TT-treated groups (Ekeuku et al., 2025). The preservation of osteocyte numbers is significant because osteocyte loss is a hallmark of postmenopausal osteoporosis, where oestrogen deficiency increases osteocyte apoptosis (McNamara, 2021).

A significantly lower *Dmp1* gene expression was noted in the TT-treated group, but no significant changes in *Sost* and *Dkk1* were observed (Ekeuku et al., 2025). The interpretation of reduced DMP1 expression is complex. The lower expression might indicate a more mature, stable osteocyte population, as DMP1 is highly expressed during osteocyte differentiation but may decrease in fully mature osteocytes (Kamiya and Takagi, 2004). The lack of changes in SOST and DKK1 expression in this study contrasts with the previous therapeutic study that showed reduced SOST expression. These observations, combined with preserved osteocyte numbers, suggest that in the preventive model, TTs primarily work by protecting osteocytes from oestrogen deficiency-induced apoptosis rather than by modulating Wnt inhibitor expression.

Metabolic syndrome has been shown to exert degenerative effects on bone health, particularly on the trabecular structures, by elevating inflammation (Wong et al., 2018a). In a study using rats with metabolic syndrome induced by high-fat high-carbohydrate diet, palm and annatto TT supplementation reduced the expression of several key osteocyte-derived factors, namely, SOST, DKK1, FGF23, and RANKL (Wong et al., 2019a).

The concurrent reduction in both SOST and DKK1 represents a coordinated decrease in Wnt pathway inhibition, which would synergistically enhance osteoblast differentiation and activity (Hu et al., 2024). FGF23 is often elevated in metabolic syndrome and obesity (Kawai, 2016). Its reduction suggests improved osteocyte metabolic health and reduced inflammatory stress. RANKL is the primary regulator of osteoclastogenesis (Weitzmann, 2013). Its reduction indicates a shift toward an anti-resorptive profile, with decreased osteoclast formation and activity. This would be expected to reduce bone resorption and help preserve bone mass.

These changes collectively enhance osteocyte-mediated signalling and shift towards an anti-resorptive profile, with reduced bone resorption (via decreased RANKL) and potentially increased bone formation (via decreased SOST and DKK1).

A subsequent study using the same metabolic syndrome model found decreased cortical osteocyte numbers, skeletal DMP1 and PHEX levels and increased empty lacunae numbers in rats with metabolic syndrome (Wong et al., 2022). These findings suggest that metabolic syndrome leads to osteocyte death and dysfunction, characterised by impaired expression of proteins crucial for bone mineralisation and phosphate metabolism.

Only annatto TT reversed the decreased empty lacunae, but palm TT did not (Wong et al., 2022). Despite these changes, DMP1 and PHEX remained unchanged with annatto or palm TT treatments (Wong et al., 2022). The differential effects between annatto and palm TTs on empty lacunae but not on DMP1/PHEX expression suggest that annatto TT may have superior antioxidant or anti-apoptotic properties that better protect osteocytes from metabolic syndrome-induced death. The composition of TT isomers differs between palm (rich in γ and α-TT) and annatto (predominantly δ-TT), which may account for functional differences. Additionally, preventing osteocyte death (by reducing empty lacunae) may not immediately restore DMP1 and PHEX expression, which might require a longer treatment duration or additional interventions.

Collectively, these studies suggest dual protective actions of TTs on bone health through osteocytes. Firstly, TTs function as potent cytoprotective agents by activating the PI3K/Akt and Nrf2 pathways, thereby preserving the integrity of the osteocyte lacunocanalicular network (Casati et al., 2020). This is particularly evident in preventive models, where early supplementation forestalls the apoptosis typically induced by oestrogen deficiency (Ekeuku et al., 2025). Secondly, TTs appear to modulate the osteocyte secretome by downregulating key Wnt inhibitors, specifically SOST and DKK1, across both established osteoporosis and metabolic syndrome models (Mohamad et al., 2021; Wong et al., 2019a). By downregulating these inhibitors, TTs facilitate the activation of Wnt/β-catenin signalling, thereby promoting an environment conducive to bone formation. Ultimately, these findings indicate that the primary bone-sparing effect of TTs may not rely solely on maintaining osteocyte numbers, but also on driving the remaining osteocytes toward a pro-anabolic and anti-resorptive profile by reducing RANKL and Wnt antagonists. The findings of these studies are summarised in Table 1.

**TABLE 1:** Summary of existing studies on the effects of TTs on osteocytes.

Study	Model	Treatment	Changes in osteocyte parameters (vs. osteoporosis control)			Remarks
			Increased	Decreased	Unchanged	
Casati et al. (2020)	MLO-Y4 cells exposed to t-BHP at 250 µM or 125 µM for 3 h	Delta-TT (1.25–20 μg/mL for 2 h) (pre-treatment)	Cell viability (1.25–10 μg/mL)	Apoptosis (5 μg/mL)	-	The redox signalling parameters were tested in osteoblasts only
Wong et al. (2019a)	Wistar rats fed with high-fat high-carbohydrate (HFHC) diet	Palm or annatto TT (60 or 100 mg/kg) (p.o.)	-	Skeletal protein expression of SOST, DKK-1, FGF23 and RANKL	Skeletal protein expression of OPG	Treatment was started 8 weeks after HFHC diet was started
Wong et al. (2022)	Wistar rats fed with high-fat high-carbohydrate (HFHC) diet	Palm or annatto TT (60 or 100 mg/kg) (p.o.)	-	Ct empty lacunae number (AnTT at 100 mg/kg)	Tb and Ct osteocyte number Tb empty lacunae number Serum and bone DMP1 protein level Bone PHEX protein level	Treatment was started 8 weeks after HFHC diet was started
Mohamad et al. (2021)	Ovariectomised female SD rats	Formulated or self-emulsified annatto TT (100 mg/kg) for 2 months (p.o.)	-	Skeletal SOST protein level	Tb osteocyte number Tb empty lacunae number Skeletal DKK1 protein level	Treatment was started 2 months after ovariectomy
Ekeuku et al. (2025)	Ovariectomised female SD rats	Formulated (100 mg/kg) or self-emulsified palm TT (50 mg/kg) for 10 weeks (p.o.)	Tb osteocyte number Ct osteocyte number	Skeletal *Dmp1* gene expression	Tb and Ct empty lacunae number Skeletal *Dkk1* and *Sost* gene expressions	n/a

Abbreviation: Ct, cortical; DKK1, dickkopf-related protein 1; DMP1, dentin matrix protein 1; FGF23, fibroblast growth factor 23; HFHC, high-fat high carbohydrate; n/a, not available; PHEX, phosphate-regulating neutral endopeptidase; p.o. oral gavage; OPG, osteoprotegerin; RANKL, receptor activator of nuclear factor κB ligand; SOST, sclerostin; Tb, trabecular; TT, tocotrienol.

These limited studies have their shortcomings. Firstly, most *in vivo* studies also use only one or two doses (60 mg/kg or 100 mg/kg), previously established to prevent bone loss in animal models (Chin, 2024b). A dose-dependent effect on osteocytes could not be established by these studies. Secondly, most studies used young and growing rats as samples. Despite being sexually mature, the skeleton of rats continues to grow until an advanced age (Fukuda and Iida, 2004). Any assault at this age (ovariectomy or diet modification) may produce stunted growth rather than degenerative changes as in osteoporosis. Thus, researchers continue to debate the relevance and representativeness of these models, despite their widespread use. Lastly, some outcomes were only measurable at the end of the experiment (e.g., osteocyte number and skeletal expression of critical markers), preventing the tracking of changes over time. Therefore, missing the window may explain the lack of changes between treatments and the osteoporosis control.

## 4 Perspectives

Given the limitations in the field, we propose several limitations for future researchers. Many studies rely on simple *in vitro* models that examine osteocytes and other bone cells separately, which can differ from the complex, coordinated bone environment in an organism. Overcoming this limitation would require a three-dimensional co-culture system or a bone explant, with mechanical loading platforms, which can help determine osteoblast migration more accurately by mimicking real bone environments. Other advancements in osteocyte culture, including osteocyte-in-chip and spheroid culture, would better mimic skeletal micro-environments and help in future investigations (Kim and Adachi, 2019; Nasello et al., 2020).

The barriers of direct observation of bone cells *in vivo* have recently been overcome with advances in microscopy. Multiphoton microscopy, when combined with genetically encoded fluorescent probes or locally injected nanoparticles (e.g., Cornell Prime Dots), enables deep tissue imaging of osteocytes in live animals, overcoming the optical scattering associated with mineralised bone (Matthews et al., 2023). Third harmonic generation microscopy, a label-free and non-invasive technique, enables high-resolution imaging of the osteocyte lacunar-canalicular network in live mice. It can distinguish osteocyte boundaries and canaliculi without the use of dyes or sectioning, and has been used to analyse osteocyte density and morphology *in vivo* (Tokarz et al., 2017). These techniques would enable the real-time tracking of TT’s action on osteocytes.

Future studies should also consider ablating the function of mature osteocytes to assess whether the bone-protective effects of TTs are retained. One example of such models is the DMP1-diphtheria toxin receptor (DMP1-DTR) mouse model. In this model, transgenic mice express the human DTR under the DMP1 promoter in osteocytes. Administration of diphtheria toxin (DT) selectively ablates 70%–80% of osteocytes *in vivo*, sparing osteoblasts (Tatsumi et al., 2007). Such a model can help to explain the role of osteocytes in TT’s skeletal action.

Furthermore, further studies should also explore how TTs coordinate multiple signalling pathways, rather than acting through a single molecular route. Fundamentally, connecting these findings to functional outcomes, such as mineralisation, perilacunar remodelling, bone strength, and phosphate regulation, will help us understand the therapeutic potential of TTs in enhancing overall bone quality.

## 5 Conclusion

In conclusion, studies have demonstrated that TTs exert protective and anabolic effects on bone health by influencing osteocytes and osteoblasts. TTs play a role in protecting osteocytes from oxidative damage and expression of their markers, but functional evidence remains lacking. Despite the established bone-protective effects of TTs on the skeletal system, further research is needed to elucidate their roles in modulating the activities of osteocytes.

## Data Availability

The original contributions presented in the study are included in the article/supplementary material, further inquiries can be directed to the corresponding authors.

## References

[B1] Abd Manan N. Mohamed N. Shuid A. N. (2012). Effects of low-dose *versus* high-dose γ-Tocotrienol on the bone cells exposed to the hydrogen peroxide-induced oxidative stress and apoptosis. Evid. Based Complement. Altern. Med. 2012, 680834. 10.1155/2012/680834 22956976 PMC3432387

[B2] Aggarwal B. B. Sundaram C. Prasad S. Kannappan R. (2010). Tocotrienols, the vitamin E of the 21st century: its potential against cancer and other chronic diseases. Biochem. Pharmacol. 80, 1613–1631. 10.1016/j.bcp.2010.07.043 20696139 PMC2956867

[B3] Ardura J. A. Álvarez-Carrión L. Gortázar A. R. Alonso V. (2020). “Chapter 6 - linking bone cells, aging, and oxidative stress: osteoblasts, osteoclasts, osteocytes, and bone marrow cells,” in Aging. Editors PREEDY V. R. PATEL V. B. , Second Edition (Academic Press).

[B4] Casati L. Pagani F. Limonta P. Vanetti C. Stancari G. Sibilia V. (2020). Beneficial effects of δ-tocotrienol against oxidative stress in osteoblastic cells: studies on the mechanisms of action. Eur. J. Nutr. 59, 1975–1987. 10.1007/s00394-019-02047-9 31280345 PMC7351870

[B5] Chen M. Li W. Lei L. Zhang L. (2025). Role of SOST in response to mechanical stimulation in bone and extraosseous organs. Biomolecules 15, 856. 10.3390/biom15060856 40563496 PMC12190277

[B6] Chin K.-Y. (2024a). “Recent progress on the skeletal research of tocotrienol,” in Lipophilic vitamins in health and disease. Editors TAPPIA P. S. SHAH A. K. DHALLA N. S. (Cham: Springer International Publishing).

[B7] Chin K. Y. (2024b). Updates in the skeletal and joint protective effects of tocotrienol: a mini review. Front. Endocrinol. (Lausanne) 15, 1417191. 10.3389/fendo.2024.1417191 38974581 PMC11224474

[B8] Chin K. Y. Mo H. Soelaiman I. N. (2013). A review of the possible mechanisms of action of tocotrienol - a potential antiosteoporotic agent. Curr. Drug Targets 14, 1533–1541. 10.2174/13894501113149990178 23859472

[B9] Chin K.-Y. Ng B. N. Rostam M. K. I. Muhammad Fadzil N. F. D. Raman V. Mohamed Yunus F. (2022). A mini review on osteoporosis: from biology to pharmacological management of bone loss. J. Clin. Med. 11, 6434. 10.3390/jcm11216434 36362662 PMC9657533

[B10] Delgado-Calle J. Sato A. Y. Bellido T. (2017). Role and mechanism of action of sclerostin in bone. Bone 96, 29–37. 10.1016/j.bone.2016.10.007 27742498 PMC5328835

[B11] Divieti Pajevic P. Krause D. S. (2019). Osteocyte regulation of bone and blood. Bone 119, 13–18. 10.1016/j.bone.2018.02.012 29458123 PMC6095825

[B12] Ekeuku S. O. Zarir S. Z. Razali A. N. Mohamad Zaidi S. Mohamed Ali Jinnah N. H. Nor Muhamad M. L. (2025). Palm tocotrienol preserves trabecular osteocyte indices and modulates the expression of osteocyte markers in ovariectomized rats. Biomedicines 13, 1220. 10.3390/biomedicines13051220 40427047 PMC12108835

[B13] Elahmer N. R. Wong S. K. Mohamed N. Alias E. Chin K.-Y. Muhammad N. (2024). Mechanistic insights and therapeutic strategies in osteoporosis: a comprehensive review. Biomedicines 12, 1635. 10.3390/biomedicines12081635 39200100 PMC11351389

[B14] Fukuda S. Iida H. (2004). Age-related changes in bone mineral density, cross-sectional area and the strength of long bones in the hind limbs and first lumbar vertebra in female wistar rats. J. Vet. Med. Sci. 66, 755–760. 10.1292/jvms.66.755 15297744

[B15] GBD 2019 FRACTURE COLLABORATORS (2021). Global, regional, and national burden of bone fractures in 204 countries and territories, 1990-2019: a systematic analysis from the global burden of disease study 2019. Lancet Healthy Longev. 2, e580–e592. 10.1016/S2666-7568(21)00172-0 34723233 PMC8547262

[B16] Hu L. Chen W. Qian A. Li Y. P. (2024). Wnt/β-catenin signaling components and mechanisms in bone formation, homeostasis, and disease. Bone Res. 12, 39. 10.1038/s41413-024-00342-8 38987555 PMC11237130

[B17] Kamiya N. Takagi M. (2004). Differential expression of dentin matrix protein 1, type I collagen and osteocalcin genes in rat developing mandibular bone. J. Mol. Histology 33, 545–552. 10.1023/a:1014955925339 12005026

[B18] Kar R. Riquelme M. A. Hua R. Jiang J. X. (2019). Glucocorticoid-induced autophagy protects osteocytes against oxidative stress through activation of MAPK/ERK signaling. JBMR Plus 3, e10077. 10.1002/jbm4.10077 31044179 PMC6478584

[B19] Karthik V. Guntur A. (2021). Energy metabolism of osteocytes. Curr. Osteoporos. Rep. 19, 444–451. 10.1007/s11914-021-00688-6 34117625 PMC8867538

[B20] Kawai M. (2016). The FGF23/Klotho axis in the regulation of mineral and metabolic homeostasis. Horm. Mol. Biol. Clin. Investig. 28, 55–67. 10.1515/hmbci-2015-0068 26943611

[B21] Kim J. Adachi T. (2019). Cell condensation triggers the differentiation of osteoblast precursor cells to osteocyte-like cells. Front. Bioeng. Biotechnol. 7, 288. 10.3389/fbioe.2019.00288 31709248 PMC6819367

[B22] Kitaura H. Marahleh A. Ohori F. Noguchi T. Shen W.-R. Qi J. (2020). Osteocyte-related cytokines regulate osteoclast formation and bone resorption. Int. J. Mol. Sci. 21, 21. [Online]. 10.3390/ijms21145169 32708317 PMC7404053

[B23] Kurpas A. Supeł K. Idzikowska K. Zielińska M. (2021). FGF23: a review of its role in mineral metabolism and renal and cardiovascular disease. Dis. Markers 2021, 8821292. 10.1155/2021/8821292 34055103 PMC8149241

[B24] Larsen M. H. Gundtoft P. H. Viberg B. (2025). High mortality among elderly with surgical treated femoral fracture in comparison to other surgical treated lower extremity fractures. A population-based register study from the Danish national patient registry. Injury 56, 112176. 10.1016/j.injury.2025.112176 39862495

[B25] Lerebours C. Buenzli P. R. (2016). Towards a cell-based mechanostat theory of bone: the need to account for osteocyte desensitisation and osteocyte replacement. J. Biomech. 49, 2600–2606. 10.1016/j.jbiomech.2016.05.012 27338526

[B26] Liang H. Chen S. Shi M. Xu J. Zhao C. Yang B. (2025). Global epidemiology and burden of osteoporosis among postmenopausal women: insights from the global burden of disease study 2021. NPJ Aging 11, 78. 10.1038/s41514-025-00269-2 40890217 PMC12402057

[B27] Liu Y. Zhao L. Li M. Gong W. Wang X. Cheng Y. (2025). Osteocytes produces RANKL *via* Wnt-TGFβ signaling axis for osteoclastogenesis. Int. J. Biol. Sci. 21, 5821–5841. 10.7150/ijbs.117481 41079923 PMC12509901

[B28] Marcucci G. Domazetovic V. Nediani C. Ruzzolini J. Favre C. Brandi M. L. (2023). Oxidative stress and natural antioxidants in osteoporosis: novel preventive and therapeutic approaches. Antioxidants 12, 373. 10.3390/antiox12020373 36829932 PMC9952369

[B29] Matthews M. D. Cook E. Naguib N. Wiesner U. B. Lewis K. J. (2023). Intravital imaging of osteocyte integrin dynamics with locally injectable fluorescent nanoparticles. Bone 174, 116830. 10.1016/j.bone.2023.116830 37327917

[B30] Mcnamara L. M. (2021). Osteocytes and estrogen deficiency. Curr. Osteoporos. Rep. 19, 592–603. 10.1007/s11914-021-00702-x 34826091

[B31] Mohamad N.-V. Ima-Nirwana S. Chin K.-Y. (2021). Self-emulsified annatto tocotrienol improves bone histomorphometric parameters in a rat model of oestrogen deficiency through suppression of skeletal sclerostin level and RANKL/OPG ratio. Int. J. Med. Sci. 18, 3665–3673. 10.7150/ijms.64045 34790038 PMC8579289

[B32] Nasello G. Alamán-Díez P. Schiavi J. Pérez M. Mcnamara L. García-Aznar J. (2020). Primary human osteoblasts cultured in a 3D microenvironment create a unique representative model of their differentiation into osteocytes. Front. Bioeng. Biotechnol. 24, 336. 10.3389/fbioe.2020.00336 32391343 PMC7193048

[B33] Notsu M. Kanazawa I. Takeno A. Tanaka K. I. Sugimoto T. (2019). Bazedoxifene ameliorates homocysteine-induced apoptosis *via* NADPH oxidase-interleukin 1β and 6 pathway in osteocyte-like cells. Calcif. Tissue Int. 105, 446–457. 10.1007/s00223-019-00580-7 31250042

[B34] Ormsby R. T. Hosaka K. Evdokiou A. Odysseos A. Findlay D. M. Solomon L. B. (2022). The effects of vitamin E analogues α-Tocopherol and γ-Tocotrienol on the human osteocyte response to ultra-high molecular weight polyethylene wear particles. Prosthesis 4, 480–489. 10.3390/prosthesis4030039

[B35] Pang K. L. Chin K. Y. (2019). The role of tocotrienol in protecting against metabolic diseases. Molecules 24, 923. 10.3390/molecules24050923 30845769 PMC6429133

[B36] Pang K. L. Low N. Y. Chin K. Y. (2020). A review on the role of denosumab in fracture prevention. Drug Des. Devel Ther. 14, 4029–4051. 10.2147/DDDT.S270829 33061307 PMC7534845

[B37] Pathak J. L. Bravenboer N. Klein-Nulend J. (2020). The osteocyte as the new discovery of therapeutic options in rare bone diseases. Front. Endocrinol., 11, 405. 10.3389/fendo.2020.00405 32733380 PMC7360678

[B38] Robling A. G. Bonewald L. F. (2020). The osteocyte: new insights. Annu. Rev. Physiol. 82, 485–506. 10.1146/annurev-physiol-021119-034332 32040934 PMC8274561

[B39] Shen L. Hu G. Karner C. (2022). Bioenergetic metabolism in osteoblast differentiation. Curr. Osteoporos. Rep. 20, 1–12. 10.1007/s11914-022-00721-2 35112289 PMC9245007

[B41] Tatsumi S. Ishii K. Amizuka N. Li M. Kobayashi T. Kohno K. (2007). Targeted ablation of osteocytes induces osteoporosis with defective mechanotransduction. Cell. Metab. 5, 464–475. 10.1016/j.cmet.2007.05.001 17550781

[B42] Tokarz D. Cisek R. Wein M. N. Turcotte R. Haase C. Yeh S.-C. A. (2017). Intravital imaging of osteocytes in mouse calvaria using third harmonic generation microscopy. PLOS ONE 12, e0186846. 10.1371/journal.pone.0186846 29065178 PMC5655444

[B43] Vázquez-Meza H. Vilchis-Landeros M. M. Vázquez-Carrada M. Uribe-Ramírez D. Matuz-Mares D. (2023). Cellular compartmentalization, glutathione transport and its relevance in some pathologies. Antioxidants 12, 834. 10.3390/antiox12040834 37107209 PMC10135322

[B44] Wan Hasan W. N. Abd Ghafar N. Chin K. Y. Ima-Nirwana S. (2018). Annatto-derived tocotrienol stimulates osteogenic activity in preosteoblastic MC3T3-E1 cells: a temporal sequential study. Drug Des. Devel Ther. 12, 1715–1726. 10.2147/DDDT.S168935 29942115 PMC6005313

[B45] Wan Hasan W. N. Chin K. Y. Abd Ghafar N. Soelaiman I. N. (2020). Annatto-derived tocotrienol promotes mineralization of MC3T3-E1 cells by enhancing BMP-2 protein expression *via* inhibiting RhoA activation and HMG-CoA reductase gene expression. Drug Des. Devel Ther. 14, 969–976. 10.2147/DDDT.S224941 32184566 PMC7060796

[B46] Weitzmann M. N. (2013). The role of inflammatory cytokines, the RANKL/OPG axis, and the immunoskeletal interface in physiological bone turnover and osteoporosis. Sci. (Cairo) 2013, 125705. 10.1155/2013/125705 24278766 PMC3820310

[B47] Wong S. K. Chin K. Y. Suhaimi F. H. Ahmad F. Ima-Nirwana S. (2018a). Effects of metabolic syndrome on bone mineral density, histomorphometry and remodelling markers in Male rats. PLoS One 13, e0192416. 10.1371/journal.pone.0192416 29420594 PMC5805301

[B48] Wong S. K. Chin K. Y. Suhaimi F. H. Ahmad F. Ima-Nirwana S. (2018b). Exploring the potential of tocotrienol from Bixa Orellana as a single agent targeting metabolic syndrome and bone loss. Bone 116, 8–21. 10.1016/j.bone.2018.07.003 29990585

[B49] Wong S. K. Chin K. Y. Ima-Nirwana S. (2019a). The effects of tocotrienol on bone peptides in a rat model of osteoporosis induced by metabolic syndrome: the possible communication between bone cells. Int. J. Environ. Res. Public Health 16. 10.3390/ijerph16183313 31505801 PMC6765824

[B50] Wong S. K. Mohamad N. V. Ibrahim N. Chin K. Y. Shuid A. N. Ima-Nirwana S. (2019b). The molecular mechanism of vitamin E as a bone-protecting agent: a review on current evidence. Int. J. Mol. Sci. 20. 10.3390/ijms20061453 30909398 PMC6471965

[B51] Wong S. K. Fikri N. I. A. Munesveran K. Hisham N. S. N. Lau S. H. J. Chin K. Y. (2022). Effects of tocotrienol on osteocyte-mediated phosphate metabolism in high-carbohydrate high-fat diet-induced osteoporotic rats. J. Funct. Foods 96, 105213. 10.1016/j.jff.2022.105213

[B52] Wu D. Li L. Wen Z. Wang G. (2023). Romosozumab in osteoporosis: yesterday, today and tomorrow. J. Transl. Med. 21, 668. 10.1186/s12967-023-04563-z 37759285 PMC10523692

[B53] Wu Y. Gan D. Liu Z. Qiu D. Tan G. Xu Z. (2025). Osteocytes: master orchestrators of skeletal homeostasis, remodeling, and osteoporosis pathogenesis. Front. Cell. Dev. Biol., 13, 1670716. 10.3389/fcell.2025.1670716 41081044 PMC12508660

[B54] Xu W. Li Y. Feng R. He P. Zhang Y. (2022). γ-Tocotrienol induced the proliferation and differentiation of MC3T3-E1 cells through the stimulation of the Wnt/β-catenin signaling pathway. Food Funct. 13, 398–410. 10.1039/d1fo02583j 34908071

[B55] Yan Y. Wang L. Ge L. Pathak J. L. (2020). Osteocyte-mediated translation of mechanical stimuli to cellular signaling and its role in bone and non-bone-related clinical complications. Curr. Osteoporos. Rep. 18, 67–80. 10.1007/s11914-020-00564-9 31953640

[B56] Ye G. Tan Z. Zheng Z. Wang D. (2025). Tocopherol inhibits bmsc ferroptosis through the mapk/nrf2 signalling pathway to delay the progression of osteonecrosis of the femoral head. Orthop. Proc. 107-B, 57. 10.1302/1358-992x.2025.7.057

[B57] Zhang Y. Yan M. Niu W. Mao H. Yang P. Xu B. (2022). Tricalcium phosphate particles promote pyroptotic death of calvaria osteocytes through the ROS/NLRP3/Caspase-1 signaling axis in amouse osteolysis model. Int. Immunopharmacol. 107, 108699. 10.1016/j.intimp.2022.108699 35305384

